# Impact of disasters on blood donation rates and blood safety: A systematic review and meta‐analysis

**DOI:** 10.1111/vox.13255

**Published:** 2022-02-15

**Authors:** Jorien Laermans, Dorien O, Emma Van den Bosch, Emmy De Buck, Veerle Compernolle, Eilat Shinar, Philippe Vandekerckhove

**Affiliations:** ^1^ Centre for Evidence‐Based Practice Belgian Red Cross Mechelen Belgium; ^2^ International Cooperation Belgian Red Cross Mechelen Belgium; ^3^ Department of Public Health and Primary Care, Faculty of Medicine KU Leuven Leuven Belgium; ^4^ Belgian Red Cross, Blood Services Mechelen Belgium; ^5^ Faculty of Medicine and Health Sciences Ghent University Ghent Belgium; ^6^ National Blood Services Magen David Adom Tel Aviv Israel; ^7^ Ben‐Gurion University of the Negev Beer Sheva Israel; ^8^ Global Advisory Panel on Corporate Governance and Risk Management of Blood Services in Red Cross and Red Crescent Societies (GAP) Perth Australia; ^9^ Belgian Red Cross Mechelen Belgium

**Keywords:** blood collection, blood safety, donor health, donor motivation, transfusion‐transmissible infections

## Abstract

**Background and Objectives:**

Timely and adequate access to safe blood forms an integral part of universal health coverage, but it may be compromised by natural or man‐made disasters. This systematic review provides an overview of the best available scientific evidence on the impact of disasters on blood donation rates and safety outcomes.

**Materials and Methods:**

Five databases (The Cochrane Library, MEDLINE, Embase, Web of Science and CINAHL) were searched until 27 March 2020 for (un)controlled studies investigating the impact of disasters on blood donation rates and/or safety. Risk of bias and overall certainty of the evidence were assessed using the Grading of Recommendations, Assessment, Development and Evaluation (GRADE) approach.

**Results:**

Eighteen observational studies were identified, providing very low certainty of evidence (due to high risk of bias, inconsistency and/or imprecision) on the impact of natural (12 studies) and man‐made/technological (6 studies) disasters. The available evidence did not enable us to form any generalizable conclusions on the impact on blood donation rates. Meta‐analyses could not detect any statistically significant changes in transfusion‐transmissible infection (TTI) rates [hepatitis B virus (HBV), hepatitis C virus (HCV), human immunodeficiency virus (HIV)‐1/2, human T‐lymphotropic virus I and II (HTLV‐I/II) and syphilis] in donated blood after a disaster, either in first‐time or repeat donors, although the evidence is very uncertain.

**Conclusion:**

The very low certainty of evidence synthetized in this systematic review indicates that it is very uncertain whether there is an association between disaster occurrence and changes in TTI rates in donated blood. The currently available evidence did not allow us to draw generalizable conclusions on the impact of disasters on blood donation rates.

**Highlights**

- It is very uncertain whether there is an association between disaster occurrence and statistically significant changes in transfusion‐transmissible infection rates [hepatitis B virus (HBV), hepatitis C virus (HCV), human immunodeficiency virus (HIV)‐1/2, human T‐lymphotropic virus I and II (HTLV‐I/II) and syphilis] in donated blood.
- The currently available evidence does not allow us to draw any generalizable conclusions on the impact of disasters on blood donation rates.

## INTRODUCTION

Transfusion of blood and blood components helps save millions of lives each year. As a result, blood and blood components are included in the Model List of Essential Medicines of the World Health Organization (WHO), which is a list of medicines that need to be available in a functioning health system at all times, in appropriate dosage forms, of assured quality and at prices individuals and the community can afford [1]. Blood services have the important task of maintaining a sufficient and safe blood supply [2]. A voluntary non‐remunerated donation system is the best way to maintain a continuous, sustainable and safe supply of blood [3]. To minimize the risk of transfusion‐transmitted infections (TTIs), a rigorous donor selection process is in place. The WHO recommends deferring high‐risk blood donors based on the results of general donor assessment, donor medical history and TTI risk assessment [2]. In addition, blood donors (or donations) should be screened for, at a minimum, the most common TTIs: human immunodeficiency virus (HIV)‐1/2, hepatitis B virus (HBV), hepatitis C virus (HCV) and syphilis.

Blood services must be prepared to respond quickly to changes in the everyday world, such as the occurrence of disasters. The United Nations Office for Disaster Risk Reduction defines a disaster as ‘a serious disruption of the functioning of a community or a society at any scale due to hazardous events interacting with conditions of exposure, vulnerability and capacity, leading to one or more of the following: human, material, economic and environmental losses and impacts’ [4]. These events are actualizations of hazards, which can be natural (e.g. seismic, meteorological, biological), man‐made (e.g. technological, inter‐human relationships such as terrorist attacks or war) or mixed (e.g. health‐related, deforestation or drought) [5].

When disaster strikes, the blood supply chain may be affected in different ways. Blood supply may be disrupted, for example, because of damaged donor centres or staffing issues, or reduced donor availability. Depending on the disaster type and magnitude, blood demand may increase as a result of higher blood transfusion requirements. Such a scenario could result in blood shortages. Conversely, altruistic responses of potential blood donors, often donating for the first time, may lead to unnecessarily high stocks of blood, which may need to be destroyed because of their limited shelf‐life [6]. Blood services should therefore have emergency plans in place to alleviate the stress in case of a disaster. The needs for blood should be assessed, and appeals to the community should be made only if absolutely necessary [7, 8]. Ideally, regular protocols for donor deferral should be maintained in order to ensure blood (component) safety. However, in response to certain types of emergencies where the risk of failure to provide blood would result in greater adverse health outcomes than the risk of issuing (partially) unscreened blood, blood services and the relevant regulatory authorities, governments and stakeholders may choose to deviate from standard procedures [9].

Currently, there is no systematic overview of the literature on the impact of disasters on blood donation rates, and in particular the number of first‐time donors. Furthermore, it is unclear whether a disaster would adversely affect the TTI rates. Therefore, we conducted a systematic review to answer the following question: ‘In (candidate) blood donors (Population), does the occurrence of a disaster (Intervention/exposure) compared to no disaster (Comparison), affect blood donation rates and/or blood safety (Outcome)?’

## MATERIALS AND METHODS

This systematic review was not prospectively registered, nor was a protocol prepared. It was carried out according to the pre‐defined methodological standards of the Centre for Evidence‐Based Practice [10]. Its reporting adheres to the PRISMA 2020 checklist (Table S1) [11].

### Eligibility criteria

#### Study design and publication type

Studies using an experimental [randomized, quasi‐ or non‐randomized controlled trials, (un)controlled before–after studies or (un)controlled interrupted time series] or observational design [cohort, case–control, (un)controlled before–after, cross‐sectional studies and (un)controlled interrupted time series] were eligible for inclusion. Other designs including computational modelling studies, case reports/series, narrative reviews and non‐original studies (e.g. editorials, book reviews, and commentaries) were excluded. Conference abstracts were included if the data were not covered by a peer‐reviewed publication. Other non‐peer‐reviewed publications and letters to the editor were excluded.

#### Population

All (candidate) whole‐blood/plasma/platelet donors visiting or contacting blood collection centres were eligible for inclusion, regardless of donor status (i.e. first‐time or repeat donors) and the blood donation system used (i.e. voluntary non‐remunerated, family/replacement or remunerated).

#### Intervention

Studies were included if they investigated the impact of any natural disaster (including, but not limited to cyclonic storms, droughts, floods, avalanches, earthquakes, landslides, tsunamis, tidal waves, acid rain, volcanic eruption, wildfires, storms, hurricanes, typhoons, blizzards, cyclones, heat waves, cold waves, extreme weather, rodent or insect infestation), technological or man‐made disaster (including, but not limited to work‐place, transport, biological accidents, acts of terrorism, warfare, armed conflicts, displacement of populations, starvation and famine) [12]. Studies were included regardless of whether they were accompanied by an active call to donate or not to donate blood (components). Studies investigating outbreaks (including epidemics and pandemics) were excluded.

#### Comparison

Studies were included if they compared donation rates and/or safety outcomes during a disaster scenario to those during a non‐disaster scenario (e.g. pre‐disaster).

#### Outcome

Studies containing quantitative data on outcomes reflecting blood donation rates and/or blood safety were eligible for inclusion.

For blood donation rates, these included the units of blood donated and the number of blood donors showing up at the blood bank either spontaneously or in response to an active call. Signs of willingness to donate blood in response to an active call to donate or an active call not to donate blood immediately (number of new donor registrations, website visits, phone calls to the blood bank centres) were also included. The number of blood units transfused, blood transfusions performed and patients requiring blood transfusion were not of interest.

Blood safety outcomes eligible for inclusion were positive screening reactivity rates or confirmed infection rates for TTIs, including all bacterial infections by blood‐borne bacteria and all viral infections transmissible through transfusion: hepatitis B virus (HBV; hepatitis B surface antigen [HBsAg] and/or anti‐hepatitis B core antibody [anti‐HBc]); hepatitis C virus (HCV; anti‐HCV antibody); human immunodeficiency virus 1 and 2 (HIV‐1/2; anti‐HIV‐1/2 antibodies, HIV p24 antigen); human T‐lymphotropic virus I and II (HTLV‐I/II; anti‐HLTV‐I/II antibodies) and syphilis (rapid plasma reagin [RPR] and/or syphilis antibody). Also, studies containing information on blood donor referral rates due to infectious diseases were included. The number of post‐transfusion TTIs in transfused patients and positive screening reactivity for non‐specific infectious disease markers (e.g. alanine aminotransferase) were not of interest.

#### Other selection criteria

No date restrictions were applied. Publications in any language were included, provided that an English abstract was available.

### Data sources and searches

Five databases were searched from the date of inception up to 27 March 2020: The Cochrane Library (both The Cochrane Database of Systematic Reviews and The Cochrane Controlled Register of Controlled Trials), MEDLINE (using the PubMed interface), Embase (using the Embase.com interface), CINAHL and Web of Science. Search strings comprising index terms and free‐text words in title or abstract were tailored to each specific database (Table S2). Furthermore, reference lists and the first 20 related citations in PubMed of the included records were scanned for additional studies.

### Study selection and data collection

A team of two reviewers (JL + DO/Luke Delfosse) independently screened titles and abstracts and subsequently full texts guided by the eligibility criteria, using EPPI‐Reviewer Web [13] and EndNote [14]. Discrepancies were resolved by discussion, and, where necessary, a third reviewer was consulted (EDB).

Data extraction was performed independently by two reviewers (JL + DO). For each study, the following data were extracted: study design, description of the population, intervention, comparison and outcome(s) of interest. In case of insufficient or ambiguous data, study authors were contacted if contact details were available.

Dichotomous outcome data were expressed as risk ratios (RRs) with a 95% confidence interval (CI). If possible, effect measures were calculated from raw data using Review Manager 5.4 [15].

### Risk of bias and GRADE

For each individual study, the quality was appraised by two reviewers independently (JL + DO). Risk of bias was assessed using the Grading of Recommendations, Assessment, Development and Evaluation (GRADE) key criteria for observational study limitations (‘Inappropriate eligibility criteria’, ‘Inappropriate methods for exposure variables’, ‘Not controlled for confounding’, ‘Incomplete or adequate follow‐up’, ‘Other limitations’) [16]. Discrepancies between both reviewers about individual assessments were resolved through discussion.

Next, GRADE was used to assess the overall certainty of the body of evidence for each outcome as ‘high’, ‘moderate’, ‘low’ or ‘very low’. Observational studies receive an initial grade of ‘low’ and subsequently can be downgraded [based on the risk of bias, imprecision, inconsistency, indirectness and publication (i.e. non‐reporting) bias] or upgraded (based on large effect, dose–response gradient and plausible confounding) [17].

### Data synthesis

If at least two studies provided data on the same outcome within the same treatment comparison, and we did not suspect large heterogeneity in outcome definitions and measurements, random effects meta‐analysis was performed using Review Manager 5.4 [15]. Heterogeneity was assessed through visual inspection of the forest plot and by using the *Χ* ^2^‐test and *I* ^2^ statistic. To investigate whether TTI rates varied with donor status (i.e. first‐time or repeat donors), subgroup analyses were performed. The threshold for statistical significance was set at 5%.

In case a meta‐analysis was not possible (i.e. data were reported by only a single study) or warranted (i.e. heterogeneity in outcome definitions was observed or suspected), outcome data were presented in a single forest plot (without calculating a total effect size) as a visual aid for result interpretation. Statistical synthesis of these results was deemed inappropriate, and no statements about the consistency of effects across studies or outcomes were made to avoid unintentional vote counting [18].

## RESULTS

### Search results

**FIGURE 1:**
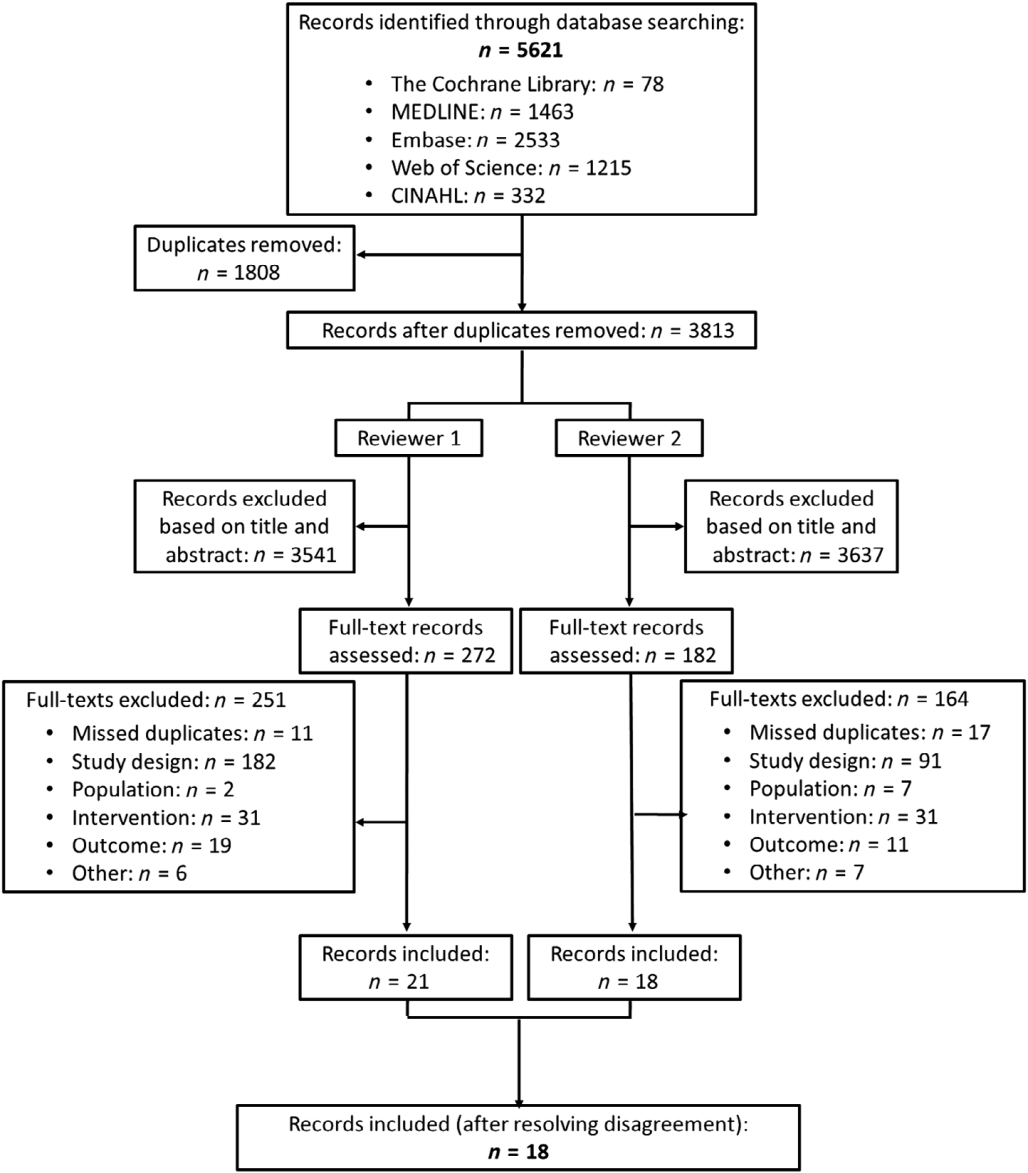
PRISMA study selection flow diagram

Figure 1 shows the detailed PRISMA study selection flow diagram. The primary searches yielded 5621 records. After removal of duplicates, titles and abstracts of the remaining 3813 records were screened. After full‐text screening and resolving disagreements, 18 records reporting on 18 unique studies were included.

### Study characteristics

A concise overview of the included studies is provided in Table 1. Detailed information on their characteristics is listed in Table S3.

**TABLE 1:** Concise overview of the included studies

Author, year, country	Disaster	Outcome measure(s)
Observational before–after studies		
Abolghasemi, 2008, Iran [19]	2003 Bam earthquake	Daily average # donations
Björk, 2017, USA [20]	2010 Haiti earthquake	# whole‐blood units collected
Busch, 1991, USA [21]	1989 San Francisco Bay earthquake	# donations collected Proportion of FTD HBsAg reactivity
Glynn, 2003, USA [22]	9/11 2001 terrorist attacks	# donations collected Proportion of FTD Weekly infectious disease marker prevalence (anti‐HIV, anti‐HCV, HBsAg) Weekly anti‐HCV prevalence
Guo, 2012, USA [23]	2008 Sichuan earthquake	# donations collected Proportion of FTD HBsAg reactivity
Hussein, 2012, Egypt [24]	Egyptian Revolution	Daily average # donations Proportion of FTD
Jalali Far, 2018, Iran [25]	2017 Kermanshah earthquake	# plasma and platelet donations collected
Kasraian, 2010, Iran [26]	2003 Bam earthquake	# donations collected Proportion of FTD HBsAg reactivity
Kuruppu, 2010, Sri Lanka [8]	2004 tsunami	# donations collected
Leung, 2019, China [27]	2017 typhoon cyclone No. 8 warning	Blood donor attendance
Lin, 2015, Taiwan [28]	2014 Kaohsiung gas explosions	# donations collected Proportion of FTD
Liu, 2010, USA [29]	2008 Sichuan earthquake	Increase in daily donations Proportion of daily donations made by FTD Overall infectious disease marker reactivity (HBsAg, anti‐HCV, anti‐HIV‐1/2, syphilis antibodies)
Rios, 2014, USA [30]	2013 Boston Marathon bombing	# donation attempts
Salah, 2018, Iran [31]	2017 Kermanshah earthquake	# donations collected Proportion of FTD Confirmed TTI seropositivity
Sönmezoglu, 2005, Turkey [32]	1999 Marmara earthquake	# donations collected Proportion of FTD HBsAg reactivity
Tran, 2010, USA [34]	9/11 2001 terrorist attacks	# donations collected Infectious disease deferral rates
Vásquez, 2011, Chile [35]	2010 Chile earthquake	# donations collected Proportion FTD
Observational cross‐sectional studies		
Spinella, 2007, USA [33]	Deployment to combat in Iraq/Afghanistan	HBsAg reactivity Anti‐HCV reactivity Anti‐HIV reactivity Anti‐HTLV‐I/II reactivity

*Note*: ‘#’ indicates ‘number of’.

*Abbreviation*: FTD, first‐time donors; HBsAg, hepatitis B surface antigen; HCV, hepatitis C virus; HIV, human immunodeficiency virus; HTLV‐I/II, human T‐lymphotropic virus I and II.

All the 18 included studies [8, 19, 20, 21, 22, 23, 24, 25, 26, 27, 28, 29, 30, 31, 32, 33, 34, 35] were observational in nature. Seventeen were uncontrolled before–after studies, whereas the 18th adopted a cross‐sectional design [33]. Eleven studies were conducted in Asia (Iran [19, 25, 26, 31], China [23, 27, 29], Iraq/Afghanistan [33], Turkey [32], Taiwan [28] and Sri Lanka [8]). The remaining seven were conducted in North America (United States [21, 22, 30, 34], Haiti [20]), South America (Chile [35]) or Africa (Egypt [24]).

Of the 12 studies providing insight into the impact of natural disasters, the vast majority (*n* = 10) reported on earthquakes hitting Iran (2003 Bam earthquake [19, 26], the 2017 Kermanshah earthquake [25, 31]), China (2008 Sichuan earthquake [23, 29]), Haiti [20], Turkey (1999 Marmara earthquake [32]), Chile (2010 earthquake [35]) and the United States (1989 San Francisco Bay earthquake [21]). The other two studies provided data on the 2004 Indian Ocean earthquake and tsunami [8] and the 2017 typhoon cyclone number 8 warning in Hong Kong [27].

Six studies contained data on the effect of man‐made or technological disasters, which included the 9/11 terrorist attacks [22, 34] and the 2013 Boston Marathon bombing [30], the 2011 three‐day Egyptian Revolution [24], the 2014 Kaohsiung gas explosions in Taiwan [28] and deployment to combat in Iraq or Afghanistan [33].

### Risk of bias and certainty of evidence

**FIGURE 2:**
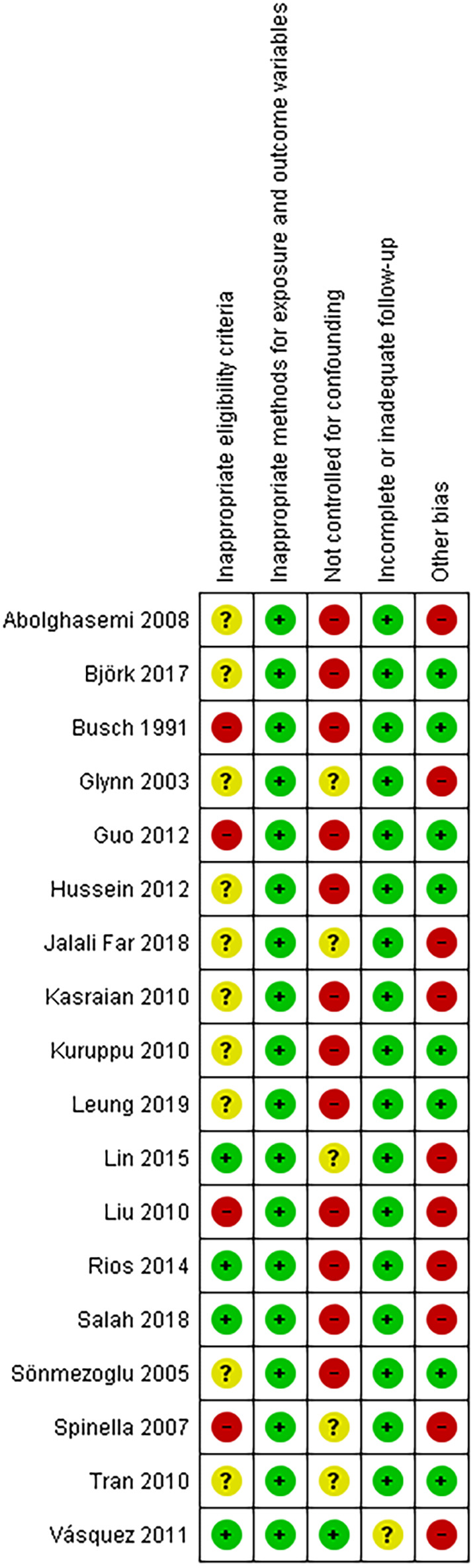
Risk of bias in the individual studies. Review authors' judgements about each risk of bias item for each included study. 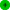 Low risk of bias. 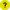 Unclear risk of bias. 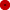 High risk of bias

The risk of bias in the individual studies is presented in Figure 2. The majority of the studies (12/18) did not adequately control their findings for confounding factors such as logistic issues (e.g. the collapse of blood collection sites during earthquakes) and differences in population demographics (e.g. more first‐time and female donors after a disaster). Four studies [21, 23, 29, 33] applied inappropriate eligibility criteria (e.g. comparing a 10‐day period after disaster to a 6‐month period before disaster), whereas 10 studies did not report sufficient information (mainly on population demographics) to make an appropriate judgement. Follow‐up was judged complete and adequate in all but one study [35]. The methods used to measure exposure and outcome variables did not raise any cause for concern in any of the studies.

Based on the risk of bias assessment, the overall certainty of the body of evidence was downgraded by one level for each outcome of interest. For the outcome of proportion of first‐time donors, the evidence was further downgraded by one level because of inconsistency due to high levels of unexplained heterogeneity. For the outcomes of HCV, HIV‐1/2 and HTLV‐I/II reactivity, the evidence was downgraded by one level because of imprecision due to the low number of events and wide 95% CIs around the effect estimates. The outcome of syphylis reactivity was downgraded by one level because of imprecision (wide 95% CIs around the effect estimates and lack of data) and by another level because of inconsistency. As a result, a very low certainty evidence level was assigned to all outcomes of interest, indicating that we are uncertain about these effect estimates.

### Synthesis of results

#### Blood donation rates

Two studies provided effect estimates on the impact of disasters on blood donation rates. In a first study, daily donations displayed a statistically significant increase of 72.6% after the 2008 Sichuan earthquake [29]. In a second study, the mean number of donation attempts was significantly lower after the Boston Marathon bombing, compared to before, but only in the group of donors who had never received a transfusion themselves [30]. The other 14 studies with data on blood donation rates after natural [8, 19, 20, 21, 23, 26, 27, 31, 32, 35] or man‐made disasters [22, 24, 28, 34] reported only absolute numbers of donations before and after the disaster. As the means and standard deviations were not available and could not be calculated, mean differences could not be estimated and statistical significance could not be judged. However, in all but three studies [8, 20, 27], the absolute number of donations after the disaster greatly exceeded the number before the disaster. The same was true for the study that provided data on plasma and platelet donation rates before and after the 2017 Kermanshah earthquake [25].

**FIGURE 3:**
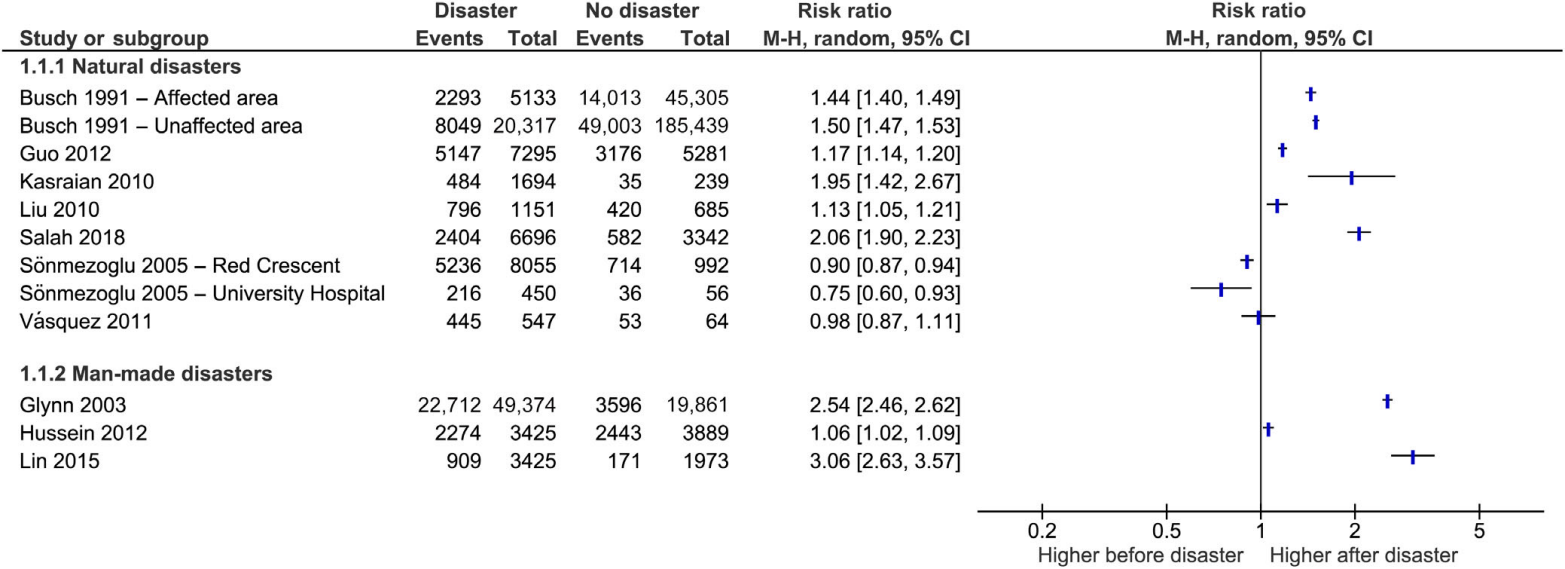
Forest plot on the proportion of first‐time donors before and after disasters. The proportion of first‐time donors presenting themselves to a blood bank in the aftermath of a disaster, compared to prior to the disaster. Events, number of first‐time donors; Total, total number of donors

Owing to very high unexplained heterogeneity (further addressed in Discussion), a meta‐analysis on the data of the effect of disasters on the proportion of first‐time donors (i.e. the number of first‐time donors divided by the total number of first‐time and repeat donors) was not warranted. Therefore, the results are presented in Figure 3 and a narrative overview is provided in the paragraphs below.

A first study showed a statistically significantly higher proportion of first‐time donors during the 10 days after the 1989 San Francisco Bay earthquake, compared to the 13‐day period before the earthquake, both in the immediately affected area of San Francisco Bay and the unaffected area of Los Angeles/Orange Counties [21]. Two studies investigating the impact of the 2008 Sichuan earthquakes revealed a statistically significant increase in the proportion of first‐time donors during the week after the earthquake, compared to the corresponding week in the following year [23], and in the proportion of daily donations made by first‐time donors during the 6‐day period after the earthquake, compared to the other 52 weeks of the same year [29]. Similarly, Kasraian et al. showed a statistically significantly higher proportion of first‐time donors during the 3‐day period following the 2003 Bam earthquake, compared to the corresponding 3 days in the previous month [26]. In the same way, there was a statistically significant increase in the proportion of first‐time donors during the 16‐day period following the 2017 Kermanshah earthquake, compared to the corresponding period of the previous year [31].

In contrast, during the 4‐day period following the 1999 Marmara earthquake, the proportion of first‐time donors was statistically significantly lower, compared to the corresponding 4‐day period in the previous year [32]. According to Vásquez et al., there was no statistically significant increase in the proportion of first‐time donors during the 5 days after the 2010 Chile earthquake, compared to the 5 days before the earthquake [35].

Following 9/11, there was a statistically significant increase in the proportion of first‐time donors during the first, second, third and fourth week after the attacks, compared to the week prior to the attacks [22]. Similarly, there was a statistically significantly higher proportion of first‐time donors during the 3‐day Egyptian Revolution, compared to the month before [24]. Finally, Lin et al. showed a statistically significantly higher proportion of first‐time donors during the first week after the 2014 Kaohsiung gas explosions, compared to the corresponding week in the previous year [28].

#### Blood safety outcomes

There is limited evidence showing a lack of association between the occurrence of a disaster and overall infectious disease marker reactivity. A meta‐analysis combining data from four studies showed that disaster occurrence was not associated with a statistically significant increase in overall infectious disease marker reactivity (Figure S1) [23, 31, 32, 34]. In an additional study, the authors failed to report the number of blood donations investigated and reported only the prevalence rates of overall infectious disease marker reactivity (i.e. HBsAg, HCV, HIV‐1/2 and syphilis) before and after the 2008 Sichuan earthquake [29]. Because of its lack of raw data, this study, in which a statistically significant increase in reactivity could not be demonstrated, was not included in the meta‐analysis. Similarly, a study reporting the weekly infectious disease marker prevalence rates (i.e. HBsAg, HCV, HIV) could not demonstrate a statistically significant increase after 9/11 [22].

In the following paragraphs, the findings for the individual TTIs (HBV, HCV, HIV‐1/2, HTLV‐I/II and syphilis) are presented.

**FIGURE 4:**
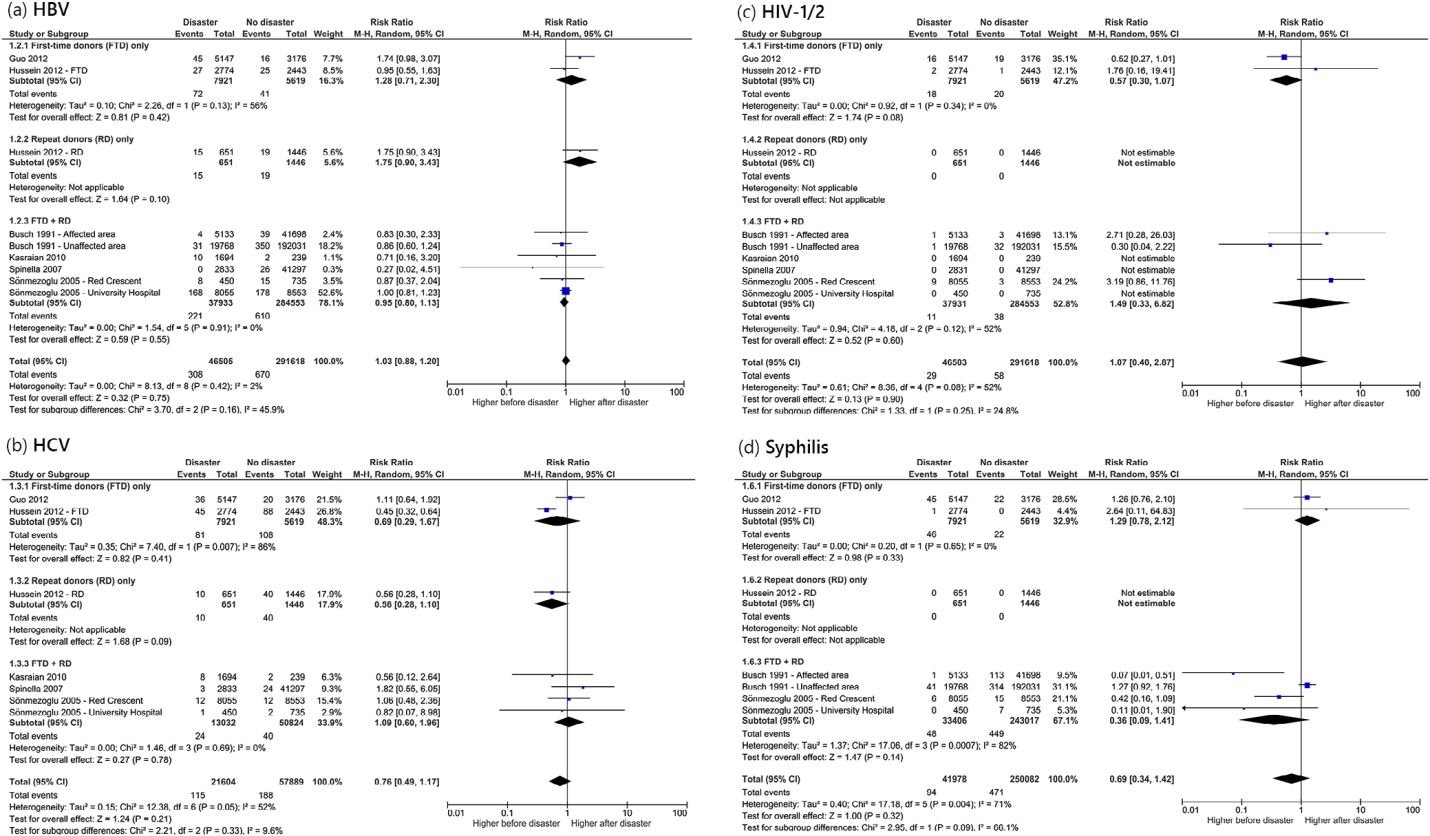
Meta‐analyses on transfusion‐transmissible infection marker reactivity rates before and after disasters. Meta‐analysis on (a) HBV, (b) HCV, (c) HIV‐1/2 and (d) syphilis marker reactivity rates in donated blood in the aftermath of a disaster, compared to prior to the disaster

*HBV*. In a meta‐analysis summarizing results of six studies, a statistically significant increase of HBsAg reactivity in case of a disaster could not be demonstrated (Figure 4a) [21, 23, 24, 26, 32, 33]. Similarly, in the 2008 Sichuan earthquake study, which was not included in the meta‐analysis, a statistically significant increase of HBsAg reactivity could not be demonstrated [29]. Similarly, another study could not detect that the 1989 San Francisco Bay earthquake was associated with a statistically significant increase of anti‐HBc reactivity in the unaffected area of Los Angeles/Orange Counties, although it did demonstrate a statistically significant increase in the immediately affected area of San Francisco Bay [21].
*HCV*. In a meta‐analysis of five studies, a statistically significant increase of anti‐HCV reactivity in case of a disaster could not be demonstrated (Figure 4b) [23, 24, 26, 32, 33]. In an additional study, which reported only the prevalence rates (therefore not included in the meta‐analysis), a statistically significant increase of anti‐HCV reactivity after the 2008 Sichuan earthquake could not be demonstrated [29]. Similarly, a study reporting weekly infectious disease marker prevalence rates could not demonstrate a statistically significant increase in anti‐HCV reactivity after 9/11 [22].
*HIV‐1/2*. In a meta‐analysis combining data from six studies, a statistically significant increase of anti‐HIV‐1/2 reactivity in case of a disaster could not be demonstrated (Figure 4c) [21, 23, 24, 26, 32, 33]. In the 2008 Sichuan earthquake study, which reported only the prevalence rates (therefore not included in the meta‐analysis), a statistically significant increase of anti‐HIV‐1/2 reactivity could not be demonstrated [29].
*HTLV‐I/II and syphilis*. In a meta‐analysis summarizing results of two studies, a statistically significant increase of anti‐HTLV‐I/II reactivity in case of a man‐made disaster could not be demonstrated (Figure S2) [21, 33].
Similarly, in a meta‐analysis of four studies, a statistically significant increase of RPR or anti‐syphilis reactivity in case of a disaster could not be demonstrated (Figure 4d) [21, 23, 24, 32]. In an additional study, which reported only the prevalence rates (therefore not included in the meta‐analysis), a statistically significant increase of anti‐syphilis reactivity after the 2008 Sichuan earthquake could not be demonstrated [29].

To investigate whether TTI reactivity rates varied with donor status, three subgroups were created: (1) first‐time donors only, (2) repeat donors only and (3) mixed donor status (first‐time + repeat). None of the performed subgroup analyses revealed between‐subgroup heterogeneity, indicating that TTI reactivity rates in first‐time donors and repeat donors are not differentially impacted by the occurrence of a disaster.

## DISCUSSION

This systematic review is the first to collect and synthetize the best available evidence on the impact of disasters on blood donation rates and blood safety outcomes, provided by 18 observational studies. Unfortunately, the available evidence did not enable us to form generalizable conclusions on the impact of disasters on blood donation rates. As for blood safety, meta‐analyses could not detect any statistically significant changes in TTI rates (HBV, HCV, HIV‐1/2, HTLV‐I/II and syphilis) in donated blood after a disaster, either in first‐time or repeat donors, although the evidence is very uncertain (all very low certainty evidence).

Altruism had been identified by previous systematic reviews as an important motivator for blood donation [36, 37, 38]. Therefore, it is not surprising that in the vast majority of the included studies, disasters were accompanied by an increase in the number of people willing to donate blood. The contrasting decreases observed in four studies might be explained by the destructive effects of the 2010 Haiti earthquake [20] and the 2004 Indian Ocean earthquake and tsunami [8] on the blood collection centres, by the suspension of the Hong Kong blood transfusion services while the typhoon cyclone No. 8 warning was in effect [27], and by the fact that the studied group of donors was confined to repeat donors who had never received a blood transfusion themselves [30]. As multiple studies indicated that the number of donations collected largely surpassed the direct needs following the disaster [6, 19, 22, 24, 29, 32, 39], blood collection services should first assess the need for blood and determine the ability to meet the demand before appealing to the community and mobilizing additional personnel. Discarding unused expired blood units is not only costly for the blood collection services but also creates a negative public image [40], and may lead to a temporary donor shortage later on because of the deferral period after a previous donation. In addition, asking donors to leave and make an appointment later that week [24] can have a negative impact on the willingness to become a regular donor, as donors feel they have been denied the opportunity to help victims.

To investigate whether a disaster coincided with a higher influx of first‐time donors, data on the proportion of first‐time donors were evaluated. Owing to considerable variation in results and inconsistency in the direction of effect among the different studies, no meta‐analysis could be performed. In exploring the causes of this heterogeneity, we considered multiple variables, including the disaster type (natural vs man‐made), whether an active call for blood donation was made or not, the blood donation system applied in the corresponding country (voluntary non‐remunerated vs remunerated vs family/replacement donation), study setting (high‐ vs low‐ and middle‐income countries) and risk of bias (high vs low/unclear). None of these variables could be identified as the (suspected) cause of this variation. Possibly, differences in a number of variables that are nearly impossible to measure objectively and accurately, such as the impact level of a certain disaster on society, lie at the root of this variation.

Our review was able to synthetize the available evidence on the influence of disasters on blood safety outcomes. Our meta‐analyses could not detect any statistically significant changes in TTI rates (HBV, HCV, HIV‐1/2, HTLV‐I/II and syphilis) in donated blood after a disaster, either in first‐time nor repeat donors. However, caution is warranted, as the results remain uncertain. This does not mean that measures taken to ensure blood safety [e.g. identification of risk behaviour in (candidate) blood donors and performing laboratory testing of all donated blood] should no longer be respected in disaster settings. Stringent measures should always stay in place to maintain the safe collection and administration of blood (components), unless absolutely necessary to meet immediate needs [9]. Moreover, especially in low‐income and lower middle‐income countries, where only 80.3% and 82% of donations are screened following basic quality procedures [41], national blood policies and legislative frameworks covering blood (component) safety should be implemented.

The current systematic review has several strengths. By searching five relevant databases and adopting comprehensive selection criteria, the review has captured outcome data on blood donation rates and blood safety, both in the absence and presence of disasters.

Nevertheless, this review also has some important limitations. Firstly, we did not consult grey literature, such as research reports and data compilations. As publishing is often not the primary activity of blood services, additional relevant data might not have been captured. Secondly, owing to the observational character of the included studies, the evidence received an initial low certainty level by default. Thirdly, thanks to the stringent blood safety measures applied by the blood services, TTI seroprevalence rates in blood donors are low, thereby hindering the precision of the results for these outcomes, which is reflected in wide confidence intervals and consequently more uncertainty about the findings. Fourthly, because of substantial risk of bias in study design and execution, the overall certainty of the evidence was judged to be very low. In addition, publication bias could not be formally tested, as the meta‐analyses performed involved only a limited number of studies. Therefore, the results of these analyses should be interpreted with caution.

In order to reach higher certainty evidence, further transparent and active reporting on the impact of disasters on blood supply and safety is warranted. At the moment, blood collection services seem to lack the reflex of publishing these readily available data in peer‐reviewed publications or share these in publicly available reports or data repositories. In addition, the current studies have failed to provide clear and quantitative information on whether they looked at voluntary non‐remunerated, family/replacement or remunerated donations (or a combination thereof). As many low‐income and lower middle‐income countries still heavily rely on family/replacement donations, or even on remunerated donations, which have been reported to be less safe than voluntary donations [3], it would be helpful to gather further evidence on the potentially differential impact of disaster occurrence across different donation systems.

On another note, in light of the global COVID‐19 pandemic, future systematic review teams may wish to consider collecting evidence on the impact of (viral or other) outbreaks on blood supply and safety. This evidence may provide useful information to blood services and national health authorities on how to develop, implement and activate emergency response plans in dealing with future pandemic outbreaks.

In conclusion, the evidence synthetized in this systematic review indicates that it is very uncertain whether the occurrence of a disaster is associated with statistically significant changed rates of TTIs in donated blood. Conclusions on the impact of disasters on blood donation rates could not be made.

## CONFLICT OF INTEREST

J.L., D.O., E.V.d.B., E.D.B., V.C. and P.V. are employees of Belgian Red Cross‐Flanders, which is responsible for supplying adequate quantities of safe blood (components) to hospitals in Flanders and Brussels on a continuous basis and is being paid for this activity by the Ministry of Social Affairs. E.S. has no conflict of interest.

## Supporting information


**Table S1** PRISMA 2020 checklist.
**Table S2** Search strings.
**Table S3** Characteristics of included studies.
**Figure S1** Meta‐analysis on overall infectious disease marker reactivity rates before and after disasters.
**Figure S2** Meta‐analysis on HTLV‐I/II reactivity rates before and after disasters.
