# Supplementary material for: Impact of disasters on blood donation rates and blood safety: A systematic review and meta‐analysis
Source: Vox Sang. 2022 Feb 15;117(6):769–79. doi: 10.1111/vox.13255 (PMC9306627; doi:10.1111/vox.13255)

# Impact of disasters on blood donation rates and blood safety: a systematic review and meta-analysis – Supporting information

### Table 1: PRISMA 2020 checklist

| **Section and Topic** | **Item #** | **Checklist item** | **Location where item is reported** |
| --- | --- | --- | --- |
| **TITLE** | | |  |
| Title | 1 | Identify the report as a systematic review. | p 1 |
| **ABSTRACT** | | |  |
| Abstract | 2 | See the PRISMA 2020 for Abstracts checklist. | p 2-3 |
| **INTRODUCTION** | | |  |
| Rationale | 3 | Describe the rationale for the review in the context of existing knowledge. | p 4-5 |
| Objectives | 4 | Provide an explicit statement of the objective(s) or question(s) the review addresses. | p 5 |
| **METHODS** | | |  |
| Eligibility criteria | 5 | Specify the inclusion and exclusion criteria for the review and how studies were grouped for the syntheses. | p 6-7 |
| Information sources | 6 | Specify all databases, registers, websites, organisations, reference lists and other sources searched or consulted to identify studies. Specify the date when each source was last searched or consulted. | p 8 |
| Search strategy | 7 | Present the full search strategies for all databases, registers and websites, including any filters and limits used. | SI Table 2 |
| Selection process | 8 | Specify the methods used to decide whether a study met the inclusion criteria of the review, including how many reviewers screened each record and each report retrieved, whether they worked independently, and if applicable, details of automation tools used in the process. | p 8 |
| Data collection process | 9 | Specify the methods used to collect data from reports, including how many reviewers collected data from each report, whether they worked independently, any processes for obtaining or confirming data from study investigators, and if applicable, details of automation tools used in the process. | p 8 |
| Data items | 10a | List and define all outcomes for which data were sought. Specify whether all results that were compatible with each outcome domain in each study were sought (e.g. for all measures, time points, analyses), and if not, the methods used to decide which results to collect. | p 7 |
|  | 10b | List and define all other variables for which data were sought (e.g. participant and intervention characteristics, funding sources). Describe any assumptions made about any missing or unclear information. | p 8 |
| Study risk of bias assessment | 11 | Specify the methods used to assess risk of bias in the included studies, including details of the tool(s) used, how many reviewers assessed each study and whether they worked independently, and if applicable, details of automation tools used in the process. | p 8-9 |
| Effect measures | 12 | Specify for each outcome the effect measure(s) (e.g. risk ratio, mean difference) used in the synthesis or presentation of results. | p 8 |
| Synthesis methods | 13a | Describe the processes used to decide which studies were eligible for each synthesis (e.g. tabulating the study intervention characteristics and comparing against the planned groups for each synthesis (item #5)). | p 9 |
|  | 13b | Describe any methods required to prepare the data for presentation or synthesis, such as handling of missing summary statistics, or data conversions. | p 9 |
|  | 13c | Describe any methods used to tabulate or visually display results of individual studies and syntheses. | p 9 |
|  | 13d | Describe any methods used to synthesize results and provide a rationale for the choice(s). If meta-analysis was performed, describe the model(s), method(s) to identify the presence and extent of statistical heterogeneity, and software package(s) used. | p 9 |
|  | 13e | Describe any methods used to explore possible causes of heterogeneity among study results (e.g. subgroup analysis, meta-regression). | p 9 |
|  | 13f | Describe any sensitivity analyses conducted to assess robustness of the synthesized results. | N/A |
| Reporting bias assessment | 14 | Describe any methods used to assess risk of bias due to missing results in a synthesis (arising from reporting biases). | N/A |
| Certainty assessment | 15 | Describe any methods used to assess certainty (or confidence) in the body of evidence for an outcome. | p 8-9 |
| **RESULTS** | | |  |
| Study selection | 16a | Describe the results of the search and selection process, from the number of records identified in the search to the number of studies included in the review, ideally using a flow diagram. | p 10 and Figure 1 |
|  | 16b | Cite studies that might appear to meet the inclusion criteria, but which were excluded, and explain why they were excluded. | N/A |
| Study characteristics | 17 | Cite each included study and present its characteristics. | p 10, Table 1 and SI Table 3 |
| Risk of bias in studies | 18 | Present assessments of risk of bias for each included study. | p 10-11 and Figure 2 |
| Results of individual studies | 19 | For all outcomes, present, for each study: (a) summary statistics for each group (where appropriate) and (b) an effect estimate and its precision (e.g. confidence/credible interval), ideally using structured tables or plots. | p 11-14 and Figures 3&4 |
| Results of syntheses | 20a | For each synthesis, briefly summarise the characteristics and risk of bias among contributing studies. | p 11-14 and Figures 3&4 |
|  | 20b | Present results of all statistical syntheses conducted. If meta-analysis was done, present for each the summary estimate and its precision (e.g. confidence/credible interval) and measures of statistical heterogeneity. If comparing groups, describe the direction of the effect. | p 11-14 and Figures 3&4 |
|  | 20c | Present results of all investigations of possible causes of heterogeneity among study results. | p 15-16 |
|  | 20d | Present results of all sensitivity analyses conducted to assess the robustness of the synthesized results. | N/A |
| Reporting biases | 21 | Present assessments of risk of bias due to missing results (arising from reporting biases) for each synthesis assessed. | N/A |
| Certainty of evidence | 22 | Present assessments of certainty (or confidence) in the body of evidence for each outcome assessed. | p 11 |
| **DISCUSSION** | | |  |
| Discussion | 23a | Provide a general interpretation of the results in the context of other evidence. | p 15 |
|  | 23b | Discuss any limitations of the evidence included in the review. | p 16-17 |
|  | 23c | Discuss any limitations of the review processes used. | p 16-17 |
|  | 23d | Discuss implications of the results for practice, policy, and future research. | p 17 |
| **OTHER INFORMATION** | | |  |
| Registration and protocol | 24a | Provide registration information for the review, including register name and registration number, or state that the review was not registered. | p 6 |
|  | 24b | Indicate where the review protocol can be accessed, or state that a protocol was not prepared. | p 6 |
|  | 24c | Describe and explain any amendments to information provided at registration or in the protocol. | N/A |
| Support | 25 | Describe sources of financial or non-financial support for the review, and the role of the funders or sponsors in the review. | p 1 |
| Competing interests | 26 | Declare any competing interests of review authors. | p 1 |
| Availability of data, code and other materials | 27 | Report which of the following are publicly available and where they can be found: template data collection forms; data extracted from included studies; data used for all analyses; analytic code; any other materials used in the review. | N/A |

*From:*  Page MJ, McKenzie JE, Bossuyt PM, Boutron I, Hoffmann TC, Mulrow CD, et al. The PRISMA 2020 statement: an updated guideline for reporting systematic reviews. BMJ 2021;372:n71. doi: 10.1136/bmj.n71

For more information, visit: <http://www.prisma-statement.org/>

### Table 2: Search strings

| **Databases** |
| --- |
| The Cochrane Library (systematic reviews and controlled trials) using the following search strategy:   1. [mh “Blood Banks”] OR [mh "Blood Donors"] OR (blood NEXT withdraw*):ti,ab,kw OR (blood NEXT don*):ti,ab,kw OR (blood NEXT bank*):ti,ab,kw OR (blood NEXT center*):ti,ab,kw OR (blood NEXT centre*):ti,ab,kw OR (blood NEXT service*):ti,ab,kw OR (transfusion NEXT service*):ti,ab,kw OR (transfusion NEXT center*):ti,ab,kw OR (transfusion NEXT centre*):ti,ab,kw 2. [mh “Disasters”] OR disast*:ti,ab,kw OR calamit*:ti,ab,kw OR cataclysm*:ti,ab,kw OR catastroph*:ti,ab,kw OR traged*:ti,ab,kw 3. [mh “Cyclonic storms”] OR [mh “Droughts”] OR [mh “Floods”] OR [mh “Avalanches”] OR [mh “Earthquakes”] OR [mh “Landslides”] OR [mh “Tsunamis”] OR [mh “Tidal waves”] OR [mh “Acid rain”] OR [mh “Volcanic eruptions”] OR [mh “Wildfires”] OR [mh “Disease outbreaks”] OR storm*:ti,ab,kw OR hurricane*:ti,ab,kw OR typhoon*:ti,ab,kw OR blizzard*:ti,ab,kw OR cyclon*:ti,ab,kw OR drought*:ti,ab,kw OR flood*:ti,ab,kw OR (heat NEXT wave*):ti,ab,kw OR (cold NEXT wave*):ti,ab,kw OR “extreme weather”:ti,ab,kw OR (extreme NEXT temperature*):ti,ab,kw OR avalanche*:ti,ab,kw OR earthquake*:ti,ab,kw OR landslide*:ti,ab,kw OR rockslide*:ti,ab,kw OR rockfall*:ti,ab,kw OR subsidence:ti,ab,kw OR (mass NEXT movement*):ti,ab,kw OR mudslide*:ti,ab,kw OR tsunami*:ti,ab,kw OR (tidal:ti,ab,kw AND wave*:ti,ab,kw) OR (acid NEXT rain*):ti,ab,kw OR (volcan*:ti,ab,kw AND erupt*:ti,ab,kw) OR fire:ti,ab,kw OR fires:ti,ab,kw OR epidemic*:ti,ab,kw OR ((parasit*:ti,ab,kw OR animal*:ti,ab,kw OR rodent*:ti,ab,kw OR rat:ti,ab,kw OR rats:ti,ab,kw OR mouse:ti,ab,kw OR mice:ti,ab,kw OR insect:ti,ab,kw OR insects:ti,ab,kw) AND (outbreak*:ti,ab,kw OR infestation*:ti,ab,kw OR infested:ti,ab,kw OR plague*:ti,ab,kw)) 4. [mh “Starvation”] OR [mh “Accidents, Occupational”] OR [mh “Accidents, Traffic”] OR [mh “Accidents, Aviation”] OR [mh “Biohazard Release”] OR [mh “Radioactive Hazard Release”] OR [mh “Structure collapse”] OR [mh “Terrorism”] OR [mh “Warfare and armed conflicts”] OR [mh “Refugees”] OR starvation:ti,ab,kw OR famine*:ti,ab,kw OR (food NEXT insecurit*):ti,ab,kw OR (“food-security” NEXT emergenc*):ti,ab,kw OR ((work:ti,ab,kw OR workplace*:ti,ab,kw OR work-related:ti,ab,kw OR industr*:ti,ab,kw OR occupat*:ti,ab,kw) AND (accident*:ti,ab,kw OR injur*:ti,ab,kw)) OR ((accident*:ti,ab,kw OR crash*:ti,ab,kw OR collision*:ti,ab,kw OR pile-up*:ti,ab,kw OR shipwreck*:ti,ab,kw OR wreck*:ti,ab,kw) AND (traffic:ti,ab,kw OR car:ti,ab,kw OR cars:ti,ab,kw OR automobil*:ti,ab,kw OR motorcycl*:ti,ab,kw OR bus:ti,ab,kw OR busses:ti,ab,kw OR train*:ti,ab,kw OR railroad*:ti,ab,kw OR airplane*:ti,ab,kw OR aircraft*:ti,ab,kw OR plane*:ti,ab,kw OR aviation:ti,ab,kw OR vehicle*:ti,ab,kw OR pedestrian*:ti,ab,kw OR ship*:ti,ab,kw)) OR aircrash*:ti,ab,kw OR ((biohazard*:ti,ab,kw OR biological*:ti,ab,kw OR chemical*:ti,ab,kw OR electric*:ti,ab,kw OR electrocut*:ti,ab,kw OR nuclear:ti,ab,kw OR radioactiv*:ti,ab,kw OR reactor*:ti,ab,kw OR radiat*:ti,ab,kw OR structur*:ti,ab,kw) AND (release*:ti,ab,kw OR accident*:ti,ab,kw OR incident*:ti,ab,kw OR destruction*:ti,ab,kw OR explosion*:ti,ab,kw OR collapse*:ti,ab,kw)) OR stampede*:ti,ab,kw OR terroris*:ti,ab,kw OR attack*:ti,ab,kw OR war:ti,ab,kw OR warfare:ti,ab,kw OR military:ti,ab,kw OR combat*:ti,ab,kw OR battle*:ti,ab,kw OR conflict*:ti,ab,kw OR genocide*:ti,ab,kw OR (displaced NEXT population*):ti,ab,kw OR “displacement of populations”:ti,ab,kw OR refugee*:ti,ab,kw 5. 2-4 OR 6. 1 AND 5 |
| MEDLINE (via PubMed interface) using the following search strategy:   1. “Blood Banks”[Mesh] OR "Blood Donors"[Mesh] OR blood withdraw*[TIAB] OR blood don*[TIAB] OR blood bank*[TIAB] OR blood center*[TIAB] OR blood centre*[TIAB] OR blood service*[TIAB] OR transfusion service*[TIAB] OR transfusion center*[TIAB] OR transfusion centre*[TIAB] 2. “Disasters”[Mesh] OR disast*[TIAB] OR calamit*[TIAB] OR cataclysm*[TIAB] OR catastroph*[TIAB] OR traged*[TIAB] 3. “Cyclonic storms”[Mesh] OR “Droughts”[Mesh] OR “Floods”[Mesh] OR “Avalanches”[Mesh] OR “Earthquakes”[Mesh] OR “Landslides”[Mesh] OR “Tsunamis”[Mesh] OR “Tidal waves”[Mesh] OR “Acid rain”[Mesh] OR “Volcanic eruptions”[Mesh] OR “Wildfires”[Mesh] OR “Disease outbreaks”[Mesh] OR storm*[TIAB] OR hurricane*[TIAB] OR typhoon*[TIAB] OR blizzard*[TIAB] OR cyclon*[TIAB] OR drought*[TIAB] OR flood*[TIAB] OR heat wave*[TIAB] OR cold wave*[TIAB] OR “extreme weather”[TIAB] OR extreme temperature*[TIAB] OR avalanche*[TIAB] OR earthquake*[TIAB] OR landslide*[TIAB] OR rockslide*[TIAB] OR rockfall*[TIAB] OR subsidence[TIAB] OR mass movement*[TIAB] OR mudslide*[TIAB] OR tsunami*[TIAB] OR (tidal[TIAB] AND wave*[TIAB]) OR acid rain*[TIAB] OR (volcan*[TIAB] AND erupt*[TIAB]) OR fire[TIAB] OR fires[TIAB] OR epidemic*[TIAB] OR ((parasit*[TIAB] OR animal*[TIAB] OR rodent*[TIAB] OR rat[TIAB] OR rats[TIAB] OR mouse[TIAB] OR mice[TIAB] OR insect[TIAB] OR insects[TIAB]) AND (outbreak*[TIAB] OR infestation*[TIAB] OR infested[TIAB] OR plague*[TIAB])) 4. “Starvation”[Mesh] OR “Accidents, Occupational”[Mesh] OR “Accidents, Traffic”[Mesh] OR “Accidents, Aviation”[Mesh] OR “Biohazard Release”[Mesh] OR “Radioactive Hazard Release”[Mesh] OR “Structure collapse”[Mesh] OR “Terrorism”[Mesh] OR “Warfare and armed conflicts”[Mesh] OR “Refugees”[Mesh] OR starvation[TIAB] OR famine*[TIAB] OR food insecurit*[TIAB] OR food-security emergenc*[TIAB] OR ((work[TIAB] OR workplace*[TIAB] OR work-related[TIAB] OR industr*[TIAB] OR occupat*[TIAB]) AND (accident*[TIAB] OR injur*[TIAB])) OR ((accident*[TIAB] OR crash*[TIAB] OR collision*[TIAB] OR pile-up*[TIAB] OR shipwreck*[TIAB] OR wreck*[TIAB]) AND (traffic[TIAB] OR car[TIAB] OR cars[TIAB] OR automobil*[TIAB] OR motorcycl*[TIAB] OR bus[TIAB] OR busses[TIAB] OR train*[TIAB] OR railroad*[TIAB] OR airplane*[TIAB] OR aircraft*[TIAB] OR plane*[TIAB] OR aviation[TIAB] OR vehicle*[TIAB] OR pedestrian*[TIAB] OR ship*[TIAB])) OR aircrash*[TIAB] OR ((biohazard*[TIAB] OR biological*[TIAB] OR chemical*[TIAB] OR electric*[TIAB] OR electrocut*[TIAB] OR nuclear[TIAB] OR radioactiv*[TIAB] OR reactor*[TIAB] OR radiat*[TIAB] OR structur*) AND (release*[TIAB] OR accident*[TIAB] OR incident*[TIAB] OR destruction*[TIAB] OR explosion*[TIAB] OR collapse*[TIAB])) OR stampede*[TIAB] OR terroris*[TIAB] OR attack*[TIAB] OR war[TIAB] OR warfare[TIAB] OR military[TIAB] OR combat*[TIAB] OR battle*[TIAB] OR conflict*[TIAB] OR genocide*[TIAB] OR displaced population*[TIAB] OR “displacement of populations”[TIAB] OR refugee*[TIAB] 5. 2-4 OR 6. Supply[TIAB] OR supplies[TIAB] OR number[TIAB] OR amount*[TIAB] OR volume*[TIAB] OR rate*[TIAB] OR availab*[TIAB] OR quantit*[TIAB] 7. “Blood safety”[Mesh] OR “Blood-borne pathogens”[Mesh] OR “Bacterial infections”[Mesh] OR “Bacteremia”[Mesh] OR “Virus diseases”[Mesh] OR infection*[TIAB] OR infectious[TIAB] OR safe*[TIAB] OR hemovigilance[TIAB] OR haemovigilance[TIAB] OR “transfusion transmissible”[TIAB] OR “transfusion transmitted”[TIAB] 8. 6-7 OR 9. 1 AND 5 AND 8 |
| Embase (via Embase.com interface) using the following search strategy:   1. ‘Blood Bank’/exp OR ‘Blood Donor’/exp OR (blood NEXT/1 withdraw*):ab,ti OR (blood NEXT/1 don*):ab,ti OR (blood NEXT/1 bank*):ab,ti OR (blood NEXT/1 center*):ab,ti OR (blood NEXT/1 centre*):ab,ti OR (blood NEXT/1 service*):ab,ti OR (transfusion NEXT/1 service*):ab,ti OR (transfusion NEXT/1 center*):ab,ti OR (transfusion NEXT/1 centre*):ab,ti 2. ‘Disaster’/exp OR disast*:ab,ti OR calamit*:ab,ti OR cataclysm*:ab,ti OR catastroph*:ab,ti OR traged*:ab,ti 3. ‘Hurricane’/exp OR ‘Drought’/exp OR ‘Flooding’/exp OR ‘Avalanche’/exp OR ‘Earthquake’/exp OR ‘Landslide’/exp OR ‘Tsunami’/exp OR ‘Acid rain’/exp OR ‘Volcano’/exp OR ‘Wildfire’/exp OR ‘Epidemic’/exp OR storm*:ab,ti OR hurricane*:ab,ti OR typhoon*:ab,ti OR blizzard*:ab,ti OR cyclon*:ab,ti OR drought*:ab,ti OR flood*:ab,ti OR (heat NEXT/1 wave*):ab,ti OR (cold NEXT/1 wave*):ab,ti OR ‘extreme weather’:ab,ti OR (extreme NEXT/1 temperature*):ab,ti OR avalanche*:ab,ti OR earthquake*:ab,ti OR landslide*:ab,ti OR rockslide*:ab,ti OR rockfall*:ab,ti OR subsidence:ab,ti OR (mass NEXT/1 movement*):ab,ti OR mudslide*:ab,ti OR tsunami*:ab,ti OR (tidal:ab,ti AND wave*:ab,ti) OR (acid NEXT/1 rain*):ab,ti OR (volcan*:ab,ti AND erupt*:ab,ti) OR fire:ab,ti OR fires:ab,ti OR epidemic*:ab,ti OR ((parasit*:ab,ti OR animal*:ab,ti OR rodent*:ab,ti OR rat:ab,ti OR rats:ab,ti OR mouse:ab,ti OR mice:ab,ti OR insect:ab,ti OR insects:ab,ti) AND (outbreak*:ab,ti OR infestation*:ab,ti OR infested:ab,ti OR plague*:ab,ti)) 4. ‘Starvation’/exp OR ‘Occupational accident’/exp OR ‘Traffic accident’/exp OR ‘Aircraft accident’/exp OR ‘Biological accident’/exp OR ‘Chemical accident’/exp OR ‘Nuclear accident’/exp OR ‘Electric accident’/exp OR ‘Electrocution’/exp OR ‘Destruction’/exp OR ‘Explosion’/exp OR ‘Radiation accident’/exp OR ‘Structure collapse’/exp OR ‘Terrorism’/exp OR ‘War’/exp OR ‘Warfare’/exp OR ‘Refugee’/exp OR starvation:ab,ti OR famine*:ab,ti OR (food NEXT/1 insecurit*):ab,ti OR (‘food-security’ NEXT/1 emergenc*):ab,ti OR ((work:ab,ti OR workplace*:ab,ti OR work-related:ab,ti OR industr*:ab,ti OR occupat*:ab,ti) AND (accident*:ab,ti OR injur*:ab,ti)) OR ((accident*:ab,ti OR crash*:ab,ti OR collision*:ab,ti OR pile-up*:ab,ti OR shipwreck*:ab,ti OR wreck*:ab,ti) AND (traffic:ab,ti OR car:ab,ti OR cars:ab,ti OR automobil*:ab,ti OR motorcycl*:ab,ti OR bus:ab,ti OR busses:ab,ti OR train*:ab,ti OR railroad*:ab,ti OR airplane*:ab,ti OR aircraft*:ab,ti OR plane*:ab,ti OR aviation:ab,ti OR vehicle*:ab,ti OR pedestrian*:ab,ti OR ship*:ab,ti)) OR aircrash*:ab,ti OR ((biohazard*:ab,ti OR biological*:ab,ti OR chemical*:ab,ti OR electric*:ab,ti OR electrocut*:ab,ti OR nuclear:ab,ti OR radioactiv*:ab,ti OR reactor*:ab,ti OR radiat*:ab,ti OR structur*) AND (release*:ab,ti OR accident*:ab,ti OR incident*:ab,ti OR destruction*:ab,ti OR explosion*:ab,ti OR collapse*:ab,ti)) OR stampede*:ab,ti OR terroris*:ab,ti OR attack*:ab,ti OR war:ab,ti OR warfare:ab,ti OR military:ab,ti OR combat*:ab,ti OR battle*:ab,ti OR conflict*:ab,ti OR genocide*:ab,ti OR (displaced NEXT/1 population*):ab,ti OR ‘displacement of populations’:ab,ti OR refugee*:ab,ti 5. 2-4 OR 6. Supply:ab,ti OR supplies:ab,ti OR number:ab,ti OR amount*:ab,ti OR volume*:ab,ti OR rate*:ab,ti OR availab*:ab,ti OR quantit*:ab,ti 7. ‘Blood safety’/exp OR ‘Bacterial infection’/exp OR ‘Bloodborne bacterium’/exp OR ‘Bacteremia’/exp OR ‘Virus infection’/exp OR infection*:ab,ti OR infectious:ab,ti OR safe*:ab,ti OR hemovigilance:ab,ti OR haemovigilance:ab,ti OR ‘transfusion transmissible’:ab,ti OR ‘transfusion transmitted’:ab,ti 8. 6-7 OR 9. 1 AND 5 AND 8 |
| **CINAHL** (via EBSCOhost interface) using the following search strategy:   1. MH “Blood Banks+” OR MH "Blood Donors+" OR TI “blood withdraw*”OR AB “blood withdraw*” OR TI “blood don*” OR AB “blood don*” OR TI “blood bank*” OR AB “blood bank*” OR TI “blood center*” OR AB “blood center*” OR TI “blood centre*” OR AB “blood centre*” OR TI “blood service*” OR AB “blood service*” OR TI “transfusion service*” OR AB “transfusion service*” OR TI “transfusion center*” OR AB “transfusion center*” OR TI “transfusion centre*” OR AB “transfusion centre*” 2. MH “Disasters+” OR TI “disast*” OR AB “disast*” OR TI “calamit*” OR AB “calamit*” OR TI “cataclysm*” OR AB “cataclysm*” OR TI “catastroph*” OR AB “catastroph*” OR TI “traged*” OR AB “traged*” 3. MH “Disease Outbreaks+” OR TI “storm*” OR AB “storm*” OR TI “hurricane*” OR AB “hurricane*” OR TI “typhoon*” OR AB “typhoon*” OR TI “blizzard*” OR AB “blizzard*” OR TI “cyclon*” OR AB “cyclon*” OR TI “drought*” OR AB “drought*” OR TI “flood*” OR AB “flood*” OR TI “heat wave*” OR AB “heat wave*” OR TI “cold wave*” OR AB “cold wave*” OR TI “extreme weather” OR AB “extreme weather” OR TI “extreme temperature*” OR AB “extreme temperature*” OR TI “avalanche*” OR AB “avalanche*” OR TI “earthquake*” OR AB “earthquake*” OR TI “landslide*” OR AB “landslide*” OR TI “rockslide*” OR AB “rockslide*” OR TI “rockfall*” OR AB “rockfall*” OR TI “subsidence” OR AB “subsidence” OR TI “mass movement*” OR AB “mass movement*” OR TI “mudslide*” OR AB “mudslide*” OR TI “tsunami*” OR AB “tsunami*” OR ((TI “tidal” OR AB “tidal”) AND (TI “wave*” OR AB “wave*”)) OR TI “acid rain*” OR AB “acid rain*” OR ((TI “volcan*” OR AB “volcan*”) AND (TI “erupt*” OR AB “erupt*”)) OR TI “fire” OR AB “fire” OR TI “fires” OR AB “fires” OR TI “epidemic*” OR AB “epidemic*” OR ((TI “parasite*” OR AB “parasite*” OR TI “animal*” OR AB “animal*” OR TI “rodent*” OR AB “rodent*” OR TI “rat” OR AB “rat” OR TI “rats” OR AB “rats” OR TI “mouse” OR AB “mouse” OR TI “mice” OR AB “mice” OR TI “insect” OR AB “insect” OR TI “insects” OR AB “insects”) AND (TI “outbreak*” OR AB “outbreak*” OR TI “infestation*” OR AB “infestation*” OR TI “infested” OR AB “infested” OR TI “plague*” OR AB “plague*”)) 4. MH “Starvation+” OR MH “Accidents, Occupational+” OR “Accidents, Traffic+” OR MH “Accidents, Aviation+” OR MH “Biohazard Release+” OR MH “Chemical Hazard Release+” OR MH “Terrorism+” OR MH “War+” OR MH “Refugees+” OR AB “starvation” OR TI “starvation” OR AB “famine*” OR TI “famine*” OR AB “food insecurit*” OR TI “food insecurit*” OR AB “food-security emergenc*” OR TI “food-security emergenc*” OR ((AB “work” OR TI “work” OR AB “workplace*” OR TI “workplace*” OR AB “work-related” OR TI “work-related” OR AB “industr*” OR TI “industr*” OR AB “occupat*” OR TI “occupat*”) AND (AB “accident*” OR TI “accident*” OR AB “injur*” OR TI “injur*”)) OR ((AB “accident*” OR TI “accident*” OR AB “crash*” OR TI “crash*” OR AB “collision*” OR TI “collision*” OR AB “pile-up*” OR TI “pile-up*” OR AB “shipwreck*” OR TI “shipwreck*” OR AB “wreck*” OR TI “wreck*”) AND (AB “traffic” OR TI “traffic” OR AB “car” OR TI “car”OR AB “cars” OR TI “cars” OR AB “automobil*” OR TI “automobil*” OR AB “motorcycl*” OR TI “motorcycl*” OR AB “bus” OR TI “bus” OR AB “busses” OR TI “busses” OR AB “train*” OR TI “train*” OR AB “railroad*” OR TI “railroad*” OR AB “airplane*” OR TI “airplane*” OR AB “aircraft*” OR TI “aircraft*” OR AB “plane*” OR TI “plane*” OR AB “aviation” OR TI “aviation” OR AB “vehicle*” OR TI “vehicle*” OR AB “pedestrian*” OR TI “pedestrian*” OR AB “ship*” OR TI “ship*”)) OR AB “aircrash*” OR TI “aircrash*” OR ((AB “biohazard*” OR TI “biohazard*” OR AB “biological*” OR TI “biological*” OR AB “chemical*” OR TI “chemical*” OR AB “electric*” OR TI “electric*” OR AB “electrocut*” OR TI “electrocut*” OR AB “nuclear” OR TI “nuclear” OR AB “radioactiv*” OR TI “radioactiv*” OR AB “reactor*” OR TI “reactor*” OR AB “radiat*” OR TI “radiat*” OR AB “structur*” OR TI “structur*”) AND (AB “release*” OR TI “release*” OR AB “accident*” OR TI “accident*” OR AB “incident*” OR TI “incident*” OR AB “destruction*” OR TI “destruction*” OR AB “explosion*” OR TI “explosion*” OR AB “collapse*” OR TI “collapse*”)) OR AB “stampede*” OR TI “stampede*” OR AB “terroris*” OR TI “terroris*” OR AB “attack*” OR TI “attack*” OR AB “war” OR TI “war” OR AB “warfare” OR TI “warfare” OR AB “military” OR TI “military” OR AB “combat*” OR TI “combat*” OR AB “battle*” OR TI “battle*” OR AB “conflict*” OR TI “conflict*” OR AB “genocide*” OR TI “genocide*” OR AB “displaced population*” OR TI “displaced population*” OR AB “displacement of populations” OR TI “displacement of populations” OR AB “refugee*” OR TI “refugee*” 5. 2-4 OR 6. 1 AND 5 |
| Web of Science using the following search strategy:   1. TS=(“blood withdraw*” OR “blood don*” OR “blood bank*” OR “blood center*” OR “blood centre*” OR “blood service*” OR “transfusion service*” OR “transfusion center*” OR “transfusion centre*”) 2. TS=(“disast*” OR “calamit*”OR “cataclysm*” OR “catastroph*” OR “traged*”) 3. TS=(“storm*” OR “hurricane*” OR “typhoon*” OR “blizzard*” OR “cyclon*” OR “drought*” OR “flood*” OR “heat wave*” OR “cold wave*” OR “extreme weather” OR “extreme temperature*” OR “avalanche*” OR “earthquake*” OR “landslide*” OR “rockslide*” OR “rockfall*” OR “subsidence” OR “mass movement*” OR “mudslide*” OR “tsunami*” OR (“tidal” AND “wave*”) OR “acid rain*” OR (“volcan*” AND “erupt*”) OR “fire” OR “fires” OR “epidemic*” OR ((“parasit*” OR “animal*” OR “rodent*” OR “rat” OR “rats” OR “mouse” OR “mice” OR “insect” OR “insects”) AND (“outbreak*” OR “infestation*” OR “infested” OR “plague*”))) 4. TS=(“starvation” OR “famine*” OR “food insecurit*” OR “food-security emergenc*” OR ((“work” OR “workplace*” OR “work-related” OR “industr*” OR “occupat*”) AND (“accident*” OR “injur*”)) OR ((“accident*” OR “crash*” OR “collision*” OR “pile-up*” OR “shipwreck*” OR “wreck*”) AND (“traffic” OR “car” OR “cars” OR “automobil*” OR “motorcycl*” OR “bus” OR “busses” OR “train*” OR “railroad*” OR “airplane*” OR “aircraft*” OR “plane*” OR “aviation” OR “vehicle*” OR “pedestrian*” OR “ship*”)) OR “aircrash*” OR ((“biohazard*” OR “biological*” OR “chemical*” OR “electric*” OR “electrocut*” OR “nuclear” OR “radioactiv*” OR “reactor*” OR “radiat*” OR “structur*”) AND (“release*” OR “accident*” OR “incident*” OR “destruction*” OR “explosion*” OR “collapse*”)) OR “stampede*” OR “terroris*” OR “attack*” OR “war” OR “warfare” OR “military” OR “combat*” OR “battle*” OR “conflict*” OR “genocide*” OR “displaced population*” OR “displacement of populations” OR “refugee*”) 5. 2-4 OR 6. TS=(“supply” OR “supplies” OR “number” OR “amount*”OR “volume*” OR “rate*” OR “availab*” OR “quantit*”) 7. TS=(“infection*” OR “infectious” OR “safe*” OR “hemovigilance” OR “haemovigilance” OR “transfusion transmissible” OR “transfusion transmitted”) 8. 6-7 OR 9. 1 AND 5 AND 8 |
| **Reference lists and related citations** |
| Included articles, retrieved with the above searches, were used to identify other studies by searching (1) reference lists and (2) 20 first related citations in MEDLINE (via PubMed interface) and Embase (via Embase.com interface). |

### Table 3: Characteristics of included studies

| **Author, year, country** | **Study design** | **Intervention/Exposure** | **Population** | **Outcome measure(s)** |
| --- | --- | --- | --- | --- |
| Abolghasemi, 2008, Iran | Observational: Uncontrolled before-after study | December 26 2003 Bam earthquake measuring 6.5 on the Richter scale | Blood donors donating blood in one of the countries’ blood collection centres either: 1) during the 4 days after the earthquake (daily average number of blood units donated: 27246); 2) during the ‘normal situation’ (daily average number of blood units donated: 3458). | Daily average number of blood donations |
| Björk, 2017, USA | Observational: Uncontrolled before-after study | January 12 2010 Haiti earthquake measuring 7.0 on the Richter scale | Blood donors donating whole blood either:  1) during the 4 months after the earthquake (Feb-May 2010); 2) during the corresponding 4-month period in 2009.  Donations were made either: - In the densely populated capital city Port-au-Prince (Feb-May 2009: 3714 units donated; Feb-May 2010: 930 units donated); - In the less populated North, Central and Southern regions of the country (Feb-May 2009: 3729 units donated; Feb-May 2010: 4189 units donated). | Number of whole blood units collected |
| Busch, 1991, USA | Observational: Uncontrolled before-after study | October 17 1989 San Francisco Bay Area earthquake  + few public requests for donation | Blood donors donating blood either:  1) during the 10 days following the earthquake;  2) during the 6 preceding months.  Donations were made either:  - In the immediately affected area of San Francisco Bay Area  (pre-earthquake: 45305 donations, 47% aged 20-39 years, 53.2% male and 46.8% female; post-earthquake: 5133 donations, 60% aged 20-39 years, 43.6% male and 56.4% female); - In the unaffected area of Los Angeles/Orange Counties (pre-earthquake: 192031 donations, 53% aged 20-39 years, 56% male and 43.8% female; post-earthquake: 20317 donations, 61% aged 20-39 years; 49.1% male and 50.7% female). | - Number of blood donations collected  - Proportion of first-time donors  - HBsAg reactivity |
| Glynn, 2003, USA | Observational:  Uncontrolled before-after study | September 11 2001 terrorist attacks + mass appeals + request not to donate | 327065 volunteer blood donors making 373628 allogeneic donations at 5 large regional US blood centres collecting about 8% of the US blood supply either:  1) During the 4 weeks after the terrorist attacks (> 50% of donations from female donors);  2) During the preceding 4 weeks;  3) During the corresponding periods in 2000 (September 12-October 9 and August 15-September 11 2000; 54% of donations from male donors). | - Number of blood donations collected  - Proportion of first-time donors  - Weekly infectious disease marker prevalence (anti-HIV, anti-HCV or HBsAg)  - Weekly anti-HCV prevalence |
| Guo, 2012, USA | Observational: Uncontrolled before-after study | May 12 2008 Earthquake measuring 8.0 on the Richter scale in the Chinese province of Sichuan + calls for blood donations | Whole-blood donors donating blood at one of 5 Chinese blood centres, either: 1) in the week following the earthquake (May 13-19 2008; n=7295, 70.6% first-time donors);  2) in the corresponding week a year later (May 13-19 2009); n=5281, 60.2% first-time donors);  3) in the entire year of 2008 (except for the week following the earthquake). | - Number of blood donations collected  - Proportion of first-time donors  - HBsAg reactivity |
| Hussein, 2012, Egypt | Observational: Uncontrolled before-after study | 3-day Egyptian Revolution (protesters demanding the overthrow of the Egyptian president Mubarak’s regime got into violent clashes with security forces) + emergency blood donation call on TV + turning away thousands of donors because of limited blood bank storage capacity | Blood donors donating blood at the non-profit independent blood bank of Cairo University Hospital, either:  1) during the Revolution (January 28-30 2011);  2) during the preceding month (December 25 2010 – January 24 2011).  Demographics: 1) Revolution donors: n=3425, 1986 male and 1439 female, 81% first-time donors, mean age 31.7±10.4 years (age range: 17-53 years);  2) Pre-revolution donors: n=3889, 3112 male and 777 female, 62.8% first-time donors, mean age 36.0±9.5 years (age range: 17-58 years). | - Daily average number of blood donations  - Proportion of first-time donors |
| Jalali Far, 2018, Iran | Observational: Uncontrolled before-after study | November 12 2017 Kermanshah earthquake measuring 7.3 on the Richter scale on the Iran-Iraq border | Blood donors donating blood, plasma or platelets at mobile and fixed collection centres of the Fars province blood transfusion service either: 1) On the day after the earthquake (blood: n=4324, 3838 male and 520 female donations; plasma and platelets: n=116 donations); 2) On the same day of the previous year (blood: n=2397, 2314 male and 100 female donations; plasma and platelets: n=33 donations). | Number of plasma and platelet donations collected |
| Kasraian, 2010, Iran | Observational: Uncontrolled before-after study | December 26 2003 earthquake measuring 6.5 on the Richter scale in Bam, (in the East of Iran).  No recruitment efforts were made. | Volunteers donating blood at the Shiraz Blood Transfusion Organisation in the South of Iran, either:  1) on the day or the 2 following days after the earthquake (December 26-28 2003) (n=1694, 1200 male and 494 female, 484 first-time donors, average age 32.8±5.6 years);  2) on the corresponding days of the previous month (November 26-28 2003) (n=239, 198 male and 41 female, 35 first-time donors, average age 23.7±4.5 years). | - Number of blood donations collected  - Proportion of first-time donors  - HBsAg reactivity |
| Kuruppu, 2010, Sri Lanka | Observational: Uncontrolled before-after study | December 26 2004 tsunami  + no call for donations + persuading donors to make their voluntary donation at another proper time | Whole blood donations made either: 1) during the 4-day period before the tsunami (December 22-25 2004);  2) during the 4-day period after the tsunami (December 27-30 2004). Donations were made either:  - In the Karapitiya Hospital Blood Centre situated in the severely affected Southern province; - At the National hospital in the capital Colombo situated in the Western province. | Number of blood donations collected |
| Leung, 2019, Hong Kong (China) | Observational: Uncontrolled before-after study | June 12 2017 typhoon cyclone No. 8 warning | Blood donors presenting at the Hong Kong Red Cross Blood Transfusion Service for blood donation: 1) during the week after the warning (June 13-19 2017); 2) on the day of the warning (June 12 2017);  3) during the preceding week (June 5-11 2017). | Blood donor attendance during 7 days |
| Lin, 2015, Taiwan | Observational: Uncontrolled before-after study | July 31 2014 gas explosions in Kaohsiung + mitigation of the volume of donors | Blood donors donating blood either: 1) during the week after the gas explosions (August 1-7 2014) (n=3425, 909 first-time donors); 2) during the corresponding week one year earlier (August 1-7 2013) (n=1973, 171 first-time donors). | - Number of blood donations collected  - Proportion of first-time donors |
| Liu, 2010, USA | Observational: Uncontrolled before-after study | May 12 2008 Earthquake measuring 8.0 on the Richter scale in the Chinese province of Sichuan + call for blood donations by the Ministry of Health | 252769 allogeneic whole blood (95%) or apheresis (5%) donations made at one of 5 Chinese blood centres, either: 1) in the week following the earthquake (6 days, including May 13 through May 18 2008) (average daily donations =1151, 459 male and 692 female, 796 first-time donors, age range: 18-55 years);  2) in the other 52 weeks of 2008 (average daily donations=685, 288 male and 397 female, 420 first-time donors, age range: 18-55 years). | - Increase in daily donations - Proportion of daily donations made by first-time donors  - Overall infectious disease marker reactivity (HBsAg, anti-HCV, anti-HIV-1/2, syphilis antibodies) |
| Rios, 2014, USA | Observational: Uncontrolled before-after study  (within subjects) | April 15 2013 Boston Marathon bombing | 429 volunteer repeat allogeneic whole-blood or apheresis double red blood cell donors (232 men and 197 women) donating at selected blood drives in  three counties of a large metropolitan area in the United States either: 1) After a local act of terrorism taking place on April 15 2013; 2) Before the local act of terrorism. | Number of donation attempts |
| Salah, 2018, Iran | Observational: Uncontrolled before-after study | November 12 2017 Earthquake measuring 7.3 on the Richter scale on the Iran-Iraq border | Blood donors donating at the Fars Blood Transfusion Service either: 1) during the 16-day period following the earthquake (November 12-28 2017) (n=6696, 13.5% female, 36% first-time, 36% lapsed and 28% repeat donors); 2) during the corresponding 16-day period in 2016 (November 12-28 2016) (n=3342, 4.5% female, 17% first-time donors). | - Number of blood donations collected  - Proportion of first-time donors  - Confirmed TTI seropositivity |
| Sönmezoglu, 2005, Turkey | Observational:  Uncontrolled before-after study | August 17 1999 Marmara earthquake  measuring 7.4 on the Richter scale in the Turkish region of Marmara  + media call for public blood donations | Blood donors donating whole blood either:  1) during the 4 days following the earthquake  (August 18-21 1999);  2) during the 6 preceding weeks (July 1-August 17 1999); 3) during the corresponding 4-day period in 1998 (August 18-21 1998); 4) during the corresponding 4-day period in 2000 (August 18-21 2000).  Donations were made either:  - At 2 regional Red Crescent Blood Banks (pre-earthquake:  88% male and 12% female, 72% first-time donors; post-earthquake:  8055 donations, 89% male and 11% female, 65% first-time donors); - At the Marmara University Hospital Blood Bank (pre-earthquake: 76% male and 24% female, 64% first-time donors; post-earthquake: 450 donations, 86% male and 14% female, 48% first-time donors). | - Number of blood donations collected  - Proportion of first-time donors  - HBsAg reactivity |
| Spinella, 2007, USA | Observational: Cross-sectional study | Deployment to combat in Iraq or Afghanistan | 2831 fresh whole blood donations of deployed donors transfused at an unspecified number of Combat Support Hospitals (CSHs) in Iraq and Afghanistan between May 2003 and February 2006. Of these 2831 units, 545 units were collected at the Baghdad CSH, where up to 460 units were pre-screened for infectious diseases before actual transfusion.  Infectious disease rates of these deployed donor units were compared to those of 41297 units of non-deployed donors. These included military and civilian donors donating fresh whole blood at the Robertson Blood Collection Centre in Texas, USA. | - HBsAg reactivity  - Anti-HCV reactivity  - Anti-HIV reactivity  - Anti-HTLV-I/II reactivity |
| Tran, 2010, USA | Observational: Uncontrolled before-after study | September 11 2001 terrorist attacks | 4594 first-time blood donors, donating allogeneic whole blood at a regional blood centre either:  1) After the attacks: between September 11 and 30 2001 (n=3315, 2141 female and 1174 male).  2) Prior to the attacks: during the corresponding period in the year 2000 (between September 11 and 30 2000) (n=1279, 771 female and 508 male).  Some of these donors returned for a second donation within the next 6 months (by March 31 2002/2001):  1) After the attacks: n=1087, 729 female and 358 male;  2) Prior to the attacks: n=427, 255 female and 172 male. | - Number of blood donations collected  - Infectious disease deferral rates |
| Vásquez, 2011, Chile | Observational: Uncontrolled before-after study | February 27 2010 earthquake measuring 8.8 on the Richter scale in central and southern Chile  + radio call for blood donations | Blood donors donating blood at the Maule Health Service Management Centre either:  1) during the first week (March 1-5, n=441) and the second week (March 8-12, n=120) following the earthquake (61% female);  2) during the preceding week (February 22-26, n=175, 37% female). | - Number of blood donations collected  - Proportion of first-time donors |

### Figure S1: Meta-analysis on overall infectious disease marker reactivity rates before and after disasters


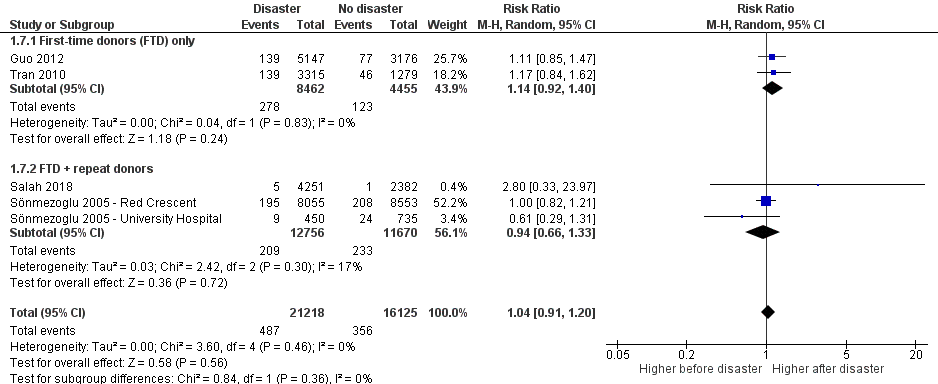


### Figure S2: Meta-analysis on HTLV-I/II reactivity rates before and after disasters


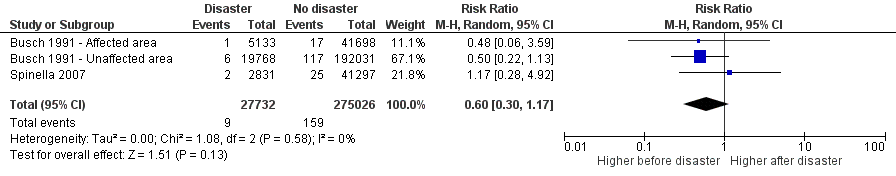

Supplement: Supplementary file 1 — Table S1 PRISMA 2020 checklist. Table S2 Search strings. Table S3 Characteristics of included studies. Figure S1 Meta‐analysis on overall infectious disease marker reactivity rates before and after disasters. Figure S2 Meta‐analysis on HTLV‐I/II reactivity rates before and after disasters. [file VOX-117-769-s001.docx]
